# Intrasession Reliability Analysis for Oscillometric Blood Pressure Method Using a Digital Blood Pressure Monitor in Peruvian Population

**DOI:** 10.3390/healthcare10020209

**Published:** 2022-01-21

**Authors:** Sabina Barrios-Fernandez, Eduardo Manuel Sosa-Sánchez, Jorge Carlos-Vivas, Laura Muñoz-Bermejo, Jesús Morenas-Martín, María Dolores Apolo-Arenas, Jose Carmelo Adsuar, Francisco Javier Domínguez-Muñoz

**Affiliations:** 1 Social Impact and Innovation in Health (InHEALTH) Research Group, Faculty of Sport Sciences, University of Extremadura, 10003 Caceres, Spain; lauramunoz@unex.es; 2 Promoting a Healthy Society Research Group (PHeSO), Faculty of Sport Sciences, University of Extremadura, 10003 Caceres, Spain; esosasan@alumnos.unex.es (E.M.S.-S.); jadssal@unex.es (J.C.A.); 3 Motor Control Research Group, Faculty of Sport Sciences, University of Extremadura, 10003 Caceres, Spain; jesusmorenas@unex.es; 4 Department of Medical and Surgical Therapeutics, Medicine and Health Sciences College, University of Extremadura, 06006 Badajoz, Spain; mdapolo@unex.es; 5 Physical Activity and Quality of Life Research Group (AFYCAV), Faculty of Sport Science, University of Extremadura, 10003 Caceres, Spain; fjdominguez@unex.es

**Keywords:** intraclass correlation coefficient, standard error of measurement, smallest real difference, blood pressure, electronic device, health promotion

## Abstract

Blood Pressure (BP) is one of the most used measured clinical parameters in health promotion and intervention. BP measures can vary due to different parameters, so we aim to study the intrasession test–retest reliability for an oscillometric method using a digital tensiometer in the Peruvian population aged over 15 with and without a diagnosis of hypertension (HT). Data were taken from the Demographic and Family Health Survey conducted in Peru in 2019. Technicians had to follow a standardized protocol on the conditions to carry out a valid and reliable measurement. Relative reliability was excellent in most cases (intraclass correlation coefficient > 0.9); absolute reliability was excellent (standard error of measurement < 5%) and smallest real difference < 10% in most cases. The Bland–Altman plot showed a systematic error of 2.36 for systolic BP in men and 2.16 in women, and 0.823 for diastolic BP in men and 0.71 for diastolic BP in women. Results suggest that the oscillometric method with a digital blood pressure monitor was reliable in absolute and relative terms in this population, so it could be used as a reliable control test to measure changes after an intervention.

## 1. Introduction

Blood pressure (BP) is created by the force of blood pushing against the walls of blood vessels as pumped by the heart [1]. BP is one of the most used measured clinical parameters in health promotion and intervention [2]. Its measurement is carried out using two numbers: systolic BP (SBP), for the pressure in the arteries during ventricular contraction, and diastolic BP (DBP), for the pressure during the ventricular relaxation phase [3]. BP normal values in adults must be under 120/80, read in millimeters of mercury (mmHg) [4]. BP varies depending on the time of day [5], body position [6] or resting state [7], so following consensus documents to carry out valid and reliable measurements is strongly recommended [8,9,10]. BP can be measured by invasive methods (arterial catheter) or with non-invasive methods, these being the most commonly used and including either manual (by auscultation or palpation) or automatic (oscillometry) methods [11].

Hypertension (HT) happens when BP force is excessive and is diagnosed if two BP readings are equal or above 130-80 on two different days, according to the American College of Cardiology and the American Heart Association guidelines published in 2017 [4]. BP can be classified into four levels: normal BP (SBP < 120 and DBP < 80 mmHg), elevated BP (SBP 120–129 and DBP < 80 mmHg), grade 1 HT (SBP 130–139 or DBP 80–89 mmHg) and grade 2 HT (SBP ≥ 140 or DBP ≥ 90 mmHg) [4,12]. HT is considered a major cause of premature death [13], being a condition that increases the risk of heart, brain, kidney, and other organs diseases, affecting 1.13 billion individuals worldwide (1/4 men and 1/5 women) according to the World Health Organization (WHO) [1]. HT family history, age, sex [14], race [15] and coexisting conditions such as diabetes or kidney diseases are non-modifiable risk factors for HT, while unhealthy diet [16], alcohol [17] and tobacco consumption [18], overweight/obesity [19,20], lack of physical activity [21] and stress [22] are considered as modifiable factors.

HT is considered a “silent killer”, so it is essential to prevent and control this condition. Health system strategies must include interventions to increase awareness, treatment and control in individuals [23]. Among non-pharmacological interventions, some recommendations for the population are carrying out a healthy lifestyle, including eating a balanced diet, avoiding alcohol and tobacco consumption, ensuring good sleep hygiene and leading a physically active lifestyle to help control weight [24]. It is also important to monitor individual through careful BP readings [25,26], ensuring that both clinicians and patients take measurements under the right conditions [27], as these may vary depending on how the readings are taken [28].

To ensure proper BP measurement, the use of a reliable device, a consistent measurement technique and the preparation of the subject in a suitable environment are required [29]. The Global Health initiative strategic plan recommends the use of an appropriate measurement protocol and proper subject preparation, the use of validated automated measurement devices and the strengthening of regulatory frameworks relevant to measurement accuracy [30]. At present, upper extremity automatic oscillometric BP measurement is preferred due to environmental issues (mercury concerns) and the risk of observer error with the auscultatory method.

The WHO recommends following a standardized procedure summarized in six steps to assure proper measurements [29,31]: (1) preparing the subject, (2) using a proper technique for BP measurement, (3) device activation following the manufacturer’s instructions, (4) recording the readings, (5) average the readings and (6) providing the reading to the patient. As stated, patient preparation and the setting are essential; the environment should be standardized and quiet, and stimuli should be avoided. Stimulant drinks, nicotine and physical activity should be avoided for the 30 min that precede the measurement. The subject should remain seated for five minutes, with legs uncrossed and feet on the floor. The cuff must be placed at heart level on the naked arm resting on the table, keeping still and quiet during the measurement [4,29,32]. Another group of important considerations are those related to the number of readings and the amount of time between the intakes. The WHO recommends taking two or more readings, separated by one–two minutes [29]. The European Society of Hypertension encourages taking three readings (two if they are normal) with one minute between readings [9,33].

Furthermore, regardless of the measurement setting (clinical or ambulatory) or the method used, the main requirement is the election of a safe and reliable device [29,34]. Lists of devices considered suitable after undergoing validation protocols from independent organizations can be found at https://www.paho.org/en/documents/lists-validated-automated-blood-pressure-measuring-devices (accessed on 3 September 2021). Test–Retest reliability is the degree to which test scores remain unchanged when measuring a stable individual characteristic on different occasions [35], ensuring the consistency of a measure, so that the proportion of the variance among scores are a result of true differences not measured differences [36].

Despite the widespread use of automatic methods for BP measurement, oscillometry must be confirmed as a reliable method with high consistency in the values obtained in repeated measurements [37]. To our knowledge, there are no publications on this issue in the general population. Therefore, we aim to determine the absolute and relative inter-session reliability of the oscillometric method using a digital BP monitor in individuals aged 15 years and older, with or without a hypertension (HTN) diagnosis, under a standardized protocol. If high inter-rater reliability of the oscillometric method is shown, it could be safely used not only in the hospital and primary care setting but also as a useful tool for home caregivers. This work may provide reliability data that is useful for establishing routine BP measurement protocols to detect and monitor potential health problems.

## 2. Materials and Methods

### 2.1. Study Design

An intrasession test–retest reliability study was conducted. The Demographic and Family Health Survey (ENDES 2019) [38] provided data to proceed with the selection of the sample. This survey was developed by the Peruvian National Institute of Statistics and Informatics (INEI). Different questionnaires were used in the survey: Household questionnaire, Women’s health questionnaire and Health questionnaire. The survey was carried out from January to December 2019. Microdata are open and available to institutions, research teams and interested stakeholders (http://iinei.inei.gob.pe/microdatos/) (accessed on 16 September 2021).

### 2.2. Participants

Thus, in the ENDES-2019 [38], residents and non-residents who spent the night at home the night before the interview aged 15 and over, participated in the survey.

Initially, there was a total sample of 34,971 participants, but data for 1449 were incomplete, so they were discarded, and the sample finally consisted of 33,522 persons: 1106 men and 1820 women with HT diagnosis; 13,133 men and 17,414 women not diagnosed; 16 men and 33 women who did not know/did not answer.

### 2.3. Ethical Considerations

Files for public use are not considered confidential, following Regulation (EU) 2016/679 of the European Parliament and of the Council of 27 April 2016 on the protection of individuals concerning the processing of personal data and on the free movement of such data (which entered into force on 25 May 2016 and has been compulsory since 25 May 2018). Data protection principles do not need to be applied to anonymous information (i.e., information related to an identifiable natural person, nor to data of subject that is not, or is no longer, identifiable). Consequently, the regulation does not affect the processing of information published by the ENDES-2019 [38]. Even for statistical or research purposes, its use does not require the approval of an accredited ethics committee.

### 2.4. Instruments and Measurements

**The Health Questionnaire** was used to collect information on the person’s history; hypertension and diabetes; risk factors for non-communicable diseases; eye and oral health; cancer prevention and control; tuberculosis; HIV/AIDS; mental health and anthropometry; BP measurements [39].

**A digital monitor of the OMRON brand** (model HEM-7113) was used for BP measurement. The selection of the model used in the oscillometric method was made by ENDES-2019 technicians based on availability at INEI. The OMRON model HEM-7113 has a digital monitor, automatic inflation by electric pump, pressure measurement range “0 to 299” mmHg, accuracy of ±3 mmHg and calibrated. Depending on the subject’s build, two types of cuffs were used, one for standard arm (220 to 320 mm) and one for wider arms (320 to 420 mm).

The BP intake was performed following a standardized protocol [40]. Instructions for the interviewers included: ensure that more than half an hour has elapsed since any stimulant drink was taken by the person; providing information about the measurement parameters, including orientation about the proper position in the chair, including keeping feet on the floor for five minutes and place right outstretched forearm with the palm facing upwards on the table; asking the individual not to move during the reading and not to speak during the reading, and to ensure that the time between the first (test) and the second intake (retest) was of two min.

### 2.5. Statistical Analysis

Statistical procedures and analysis were conducted using the Statistical Package for the Social Sciences (SPSS, Version 25, IBM SPSS, Armonk, NY, USA) software. A paired samples *t*-test was used to examine differences between both values (test and retest). Relative reliability was carried out using the Intraclass Correlation Coefficient (ICC) with 95% confidence interval across the two measurements. ICC interpretation was performed according to Munro’s criteria: 0.50–0.69 moderate, 0.70–0.89 high and >0.90 excellent [41]. Absolute reliability was determined with the Standard Error of Measurement (SEM) and the Smallest Real Difference (SRD) scores at 95% confidence interval (SRD_95_) following the equation: SEM = SD $\sqrt{(1-ICC})$, where SD (standard deviation) is the mean of the test and the retest, while ICC represents the reliability coefficient [42]. SRD_95_ = 1.96 √2SEM. The 1.96 in the SRD_95_ equation represents the z-score at the 95% confidence level. Bland–Altman graphics were shown to assess systematic error [43].

## 3. Results

Results are presented in two sections: systolic and diastolic BP reliability. In both, relative and absolute reliability values can be found, also providing information according to sex and age ranges, followed by Bland–Altman’s graphs.

### 3.1. Systolic Blood Pressure Reliability

Table 1 shows the means and standard deviations of both measurements, with 2 min in between. It also includes the *p* value, which indicates the statistically significant differences between both measures.

Table 2 shows relative and absolute reliability indices for the overall sample for systolic BP reliability. The ICC value is considered “excellent” according to Munro’s criteria [41].

Table 3 shows the means and standard deviations of both measurements, with 2 min in between. It also includes the *p* value, which indicates the statistically significant differences between both measures. It can be observed that these differences exist for both measurements, for men with and without hypertension diagnosis.

Relative and absolute reliability indices for men, with and without HT diagnosis, and grouped by age range, can be found in Table 4.

Table 5 shows the means and standard deviations of both measurements, with 2 min in between. It also includes the *p* value, which indicates the statistically significant differences between both measures. It can be observed that these differences exist for both measurements, for women with and without hypertension diagnosis.

Relative and absolute reliability indices for women, with and without HT diagnosis, and grouped by age range, are displayed in Table 6.

**Figure 1:**
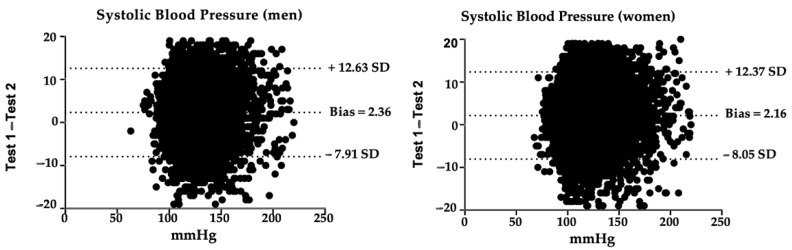
Bland–Altman graphs of the differences between test and retest systolic BP measurements. SD: standard deviation; mmHg: millimeters of mercury.

Figure 1 shows Bland–Altman graphs [43] of the differences between test and retest systolic BP measurements.

### 3.2. Diastolic Blood Pressure Reliability

Table 7 shows the means and standard deviations of both measurements, with 2 min in between. It also includes the *p* value, which indicates the statistically significant differences between both measures.

Table 8 illustrates relative and absolute reliability indices for the overall sample for diastolic BP reliability. Again, the ICC value is considered “excellent” [41].

Table 9 shows the means and standard deviations of both measurements, with 2 min in between. It also includes the *p* value, which indicates the statistically significant differences between both measures. It can be observed that these differences exist for both measurements, for men with and without hypertension diagnosis.

Table 10 shows relative and absolute reliability indices for men, with and without HT diagnosis, and grouped by age range groups.

Table 11 shows the means and standard deviations of both measurements, with 2 min in between. It also includes the *p* value, which indicates the statistically significant differences between both measures. It can be observed that these differences exist for both measurements, for women with and without hypertension diagnosis.

Table 12 shows relative and absolute reliability indices for women, with and without HT diagnosis, grouped by age.

**Figure 2:**
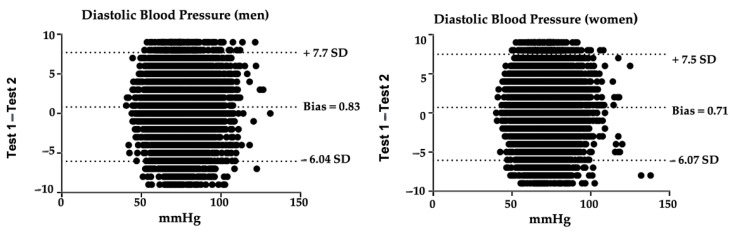
Bland–Altman graphs of the differences between test and retest diastolic BP measurements. SD: standard deviation; mmHg: millimeters of mercury.

Bland–Altman graphs [43] of the differences between test and retest diastolic BP measurements can be observed in Figure 2.

## 4. Discussion

The main finding of this work is that the oscillometric method using an OMRON digital sphygmomanometer (model HEM-7113), together with the measurement conditions set by the survey manual created by the Peruvian government [39,40], has shown high reliability in both sexes and the whole age range over 15 years in the Peruvian population (high and excellent values).

The oscillometric method has been the subject of several studies, as nowadays it is widely used [44]. Our results demonstrate high reliability in people over 15 years of age. However, automatic devices using oscillometry have also proven to be a suitable alternative for initial BP screening in children [45]. In addition, this method has proven to be reliable in patients with chronic coronary pathologies as no significant differences were found between repeated systolic and diastolic pressure measurements in patients with atrial fibrillation [46]. Other studies have carried out comparisons between the auscultation method, the automatic device and the invasive method, the conclusion of which has shown that the difference between a measurement with a digital oscillometric device compared to auscultation was less than 5 mmHg in older people with atrial fibrillation, which is considered an acceptable margin in terms of reliability. However, compared with invasive methods, the difference was <5 mmHg, which would not be clinically acceptable [47].

Regarding the reliability of the method when adopting different postures during the measurement, orthostasis for five minutes on a modified tilt table on different days showed high and excellent CCI results in both supine and tilt positions [48]. In individuals over 50 years of age, BP measurement by oscillometry in different postures and fasting states reported high and excellent values [49]. Other studies have examined the reliability of BP digital monitors for physical activity prescription. The results showed high and excellent ICC in healthy subjects performing resistance training on different days [50]. They also showed a high ICC and excellent measurement of the reliability of the automatic method in healthy individuals when performing submaximal exercise [51].

Some studies have questioned the BP monitoring devices’ reliability. In this line, when exploring the level of agreement between BP measurements in overweight/obese children using the auscultatory method (mercury sphygmomanometer) and an oscillometric device, lower systolic and diastolic BP readings were obtained with the auscultatory method than with oscillometry. Furthermore, the CCI values ranged from moderate reliability for systolic (0.595) to poor reliability for diastolic readings (0.33). Thus, due to both low reliability and large discrepancies, it is concluded that both devices should not be used equivalently [52]. Furthermore, in patients with coronary pathologies such as atrial fibrillation and HTN, it was observed that the digital oscillometric device obtained, on average, higher systolic BP values (5.3–6.3 mmHg), so a wide range of random error rates may be occurring [53].

Furthermore, in our study, the Bland–Altman graphs for both systolic and diastolic BP indicate a positive bias, which means that, in most cases, values decrease in the retest. A possible explanation for this could be the so-called white coat HT: some individuals present a variation in BP levels when they are being measured [54,55]. These individuals may be advised to self-monitor their BP at home [56], so good reliability and ease of use of digital BP digital monitors are necessary. However, Casiglia et al. warn that wrist devices for home self-measurement could lead to falsely elevated readings: although participants were trained on how to perform the measurement correctly, these errors could be due to an incorrect forearm position, so health education must therefore be considered as a fundamental pillar [57].

Several protocols for BP assessment in the monitoring of healthy and hypertensive individuals are proposed by different scientific societies at both national and international levels, established by consensus [4,12,58]. The protocol followed in BP measurement at ENDES 2019 [38], which conforms to WHO requirements, is reliable and therefore safe for establishing reliable values of the force exerted by the blood in the brachial artery.

In summary, reliability studies of digital oscillometry in both central [51,59] and peripheral [46] pressure measurements have been performed, comparing different protocols [52], in children [45,60], adults [61] and elderly people [49], in people with atrial fibrillation [46,47,53] and people undergoing endurance training [50] or with orthostatic conditions [48], all with different results (reliability between acceptable and excellent). However, high and excellent values of absolute and relative inter-session reliability of the oscillometric method using a digital BP monitor under a standardized protocol in a general population over 15 years of age will undoubtedly have a positive impact on the safety of brachial blood pressure monitoring in both hospital and home settings.

The main strengths of this study are the size of the sample and the fact that a nationally representative survey has been used. The use of the Demographic and Family Health Survey (ENDES 2019) provides representative data on the health of the population over 15 years of age and the figures from two consecutive BP readings of 33,522 individuals. These results make it possible to identify BP levels in younger individuals as well as in older age groups and to know the reliability of the method used. However, as in all studies, it also has some limitations: (1) The lack of control over the sample, since the participants are drawn from the open database of the survey ENDES 2019 [38], means we cannot be sure that the conditions during the measurements were stable in all cases. (2) Although these results can be used to determine the reliability of the automatic BP measurement device, their generalizability is limited to populations with similar characteristics and cannot be generalised to other populations (elderly, specific pathologies, etc.). (3) The sample selection did not include the group of children and adolescents, and (4) since this is a cross-sectional study and not an intervention study, it is not possible to establish cause–effect relationships.

## 5. Conclusions

Measurement of BP using a digital monitor based on the oscillometric method in conjunction with a standardized protocol is reliable for both SBP and DBP, regardless of whether individuals are healthy or diagnosed with HTN. Therefore, the protocol followed in the measurement using an automatic oscillometric device is a useful, low-cost instrument and ensures the reliability of BP measurements in healthcare and home settings. This study provides indicators that allow health professionals to monitor blood pressure in a general population with confidence.

## Figures and Tables

**Table 1:** MmHg in the digital monitor of the OMRON brand to measure the systolic blood pressure in 2 measurements with an interval of 2 min.

		Measurement 1 ± TD	Measurement 2 ± TD	*p* *
All (*n* = 33,522)	Systolic blood pressure (mmHg)	120.31 ± 15.56	118.07 ± 17.17	<0.001

mmHg: millimeters of mercury; TD: Typical Deviation; * *p* values were calculated through paired samples test.

**Table 2:** Systolic blood pressure test–retest reliability of two measurements taken with a 2-min interval (*n* = 33,522).

ICC (95% CI)	SEM (mmHg)	%SEM	SRD (mmHg)	%SRD
0.95 (0.94–0.95)	3.99	3.35	11.07	9.28

ICC: intraclass correlation coefficient; CI: confidence interval; SEM: standard error of measurement; SRD: smallest real difference; mmHg: millimeters of mercury.

**Table 3:** MmHg in the digital monitor of the OMRON brand to measure the systolic blood pressure in 2 measurements with an interval of 2 min.

		Measurement 1 ± TD	Measurement 2 ± TD	*p* *
**Men with Hypertension diagnosis (*n* = 1106)**				
15–17 (*n* = 6)	Systolic blood pressure (mmHg)	119.83 ± 5.88	119.33 ± 4.97	0.768
18–29 (*n* = 48)	Systolic blood pressure (mmHg)	127.06 ± 15.48	125.27 ± 15.72	0.021
30–39 (*n* = 107)	Systolic blood pressure (mmHg)	131.10 ± 15.42	127.66 ± 14.53	<0.001
40–49 (*n* = 181)	Systolic blood pressure (mmHg)	132.82 ± 20.24	130.77 ± 18.87	<0.001
50–59 (*n* = 199)	Systolic blood pressure (mmHg)	140.98 ± 22.71	138.65 ± 21.73	<0.001
60–69 (*n* = 220)	Systolic blood pressure (mmHg)	143.34 ± 23.36	140.35 ± 23.78	<0.001
70–79 (*n* = 220)	Systolic blood pressure (mmHg)	147.50 ± 25.16	144.90 ± 24.44	<0.001
80–89 (*n* = 114)	Systolic blood pressure (mmHg)	151.02 ± 26.50	147.63 ± 26.80	<0.001
>90 (*n* = 11)	Systolic blood pressure (mmHg)	142.91 ± 15.37	142.64 ± 17.88	0.885
**Men without Hypertension diagnosis (*n* = 13,133)**				
15–17 (*n* = 876)	Systolic blood pressure (mmHg)	118.76 ± 12.97	116.19 ± 11.81	<0.001
18–29 (*n* = 3210)	Systolic blood pressure (mmHg)	123.09 ± 11.81	120.74 ± 11.37	<0.001
30–39 (*n* = 3535)	Systolic blood pressure (mmHg)	123.67 ± 11.99	121.41 ± 11.70	<0.001
40–49 (*n* = 2208)	Systolic blood pressure (mmHg)	125.37 ± 14.17	123.34 ± 13.82	<0.001
50–59 (*n* = 1479)	Systolic blood pressure (mmHg)	127.86 ± 16.28	125.21 ± 15.67	<0.001
60–69 (*n* = 1012)	Systolic blood pressure (mmHg)	131.23 ± 19.44	128.79 ± 18.91	<0.001
70–79 (*n* = 541)	Systolic blood pressure (mmHg)	134.59 ± 21.08	132.32 ± 20.73	<.001
80–89 (*n* = 240)	Systolic blood pressure (mmHg)	132.76 ± 21.59	130.06 ± 21.13	<0.001
>90 (*n* = 32)	Systolic blood pressure (mmHg)	137.06 ± 26.83	132.56 ± 28.69	<0.001

mmHg: millimeters of mercury; TD: Typical Deviation; * *p* values were calculated through paired samples test.

**Table 4:** Relative and absolute reliability indices for men with and without a diagnosis of HT.

Age Intervals (years)	ICC (95% CI)	SEM (mmHg)	%SEM	SRD (mmHg)	%SRD
**Men with Hypertension diagnosis (*n* = 1106)**					
15–17 (*n* = 6)	0.77 (0.13–0.96)	2.58	2.16	7.716	5.99
18–29 (*n* = 48)	0.94 (0.89–0.96)	3.85	3.05	10.68	8.47
30–39 (*n* = 107)	0.91 (0.89–0.94)	4.37	3.38	12.10	9.36
40–49 (*n* = 181)	0.95 (0.94–0.96)	4.24	3.22	12	8.92
50–59 (*n* = 199)	0.96 (0.95–0.97)	4.50	3.22	12.47	8.92
60–69 (*n* = 220)	0.96 (0.95–0.97)	4.66	3.28	12.90	9.10
70–79 (*n* = 220)	0.95 (0.94–0.96)	5.26	3.60	14.58	9.97
80–89 (*n* = 114)	0.96 (0.95–0.97)	5.14	3.45	14.29	9.55
>90 (*n* = 11)	0.94 (0.80–0.98)	4.11	2.88	11.38	7.97
**Men without Hypertension diagnosis (*n* = 13,133)**					
15–17 (*n* = 876)	0.89 (0.86–0.89)	4.17	3.55	11.56	9.84
18–29 (*n* = 3210)	0.89 (0.88–0.90)	3.81	3.12	10.56	8.66
30–39 (*n* = 3535)	0.90 (0.89–0.90)	3.76	3.07	10.43	8.52
40–49 (*n* = 2208)	0.93 (0.92–0.93)	3.81	3.06	10.55	8.49
50–59 (*n* = 1479)	0.93 (0.92–0.94)	4.20	3.32	11.63	9.19
60–69 (*n* = 1012)	0.94 (0.94–0.95)	4.58	3.52	12.69	9.76
70–79 (*n* = 541)	0.95 (0.94–0.96)	4.77	3.57	13.21	9.9
80–89 (*n* = 240)	0.95 (0.94–0.96)	4.63	3.52	12.84	9.77
<90 (*n* = 32)	0.96 (0.93–0.98)	5.19	3.85	14.39	10.68

ICC: intraclass correlation coefficient; CI: confidence interval; SEM: standard error of measurement; SRD: smallest real difference; mmHg: millimeters of mercury.

**Table 5:** MmHg in the digital monitor of the OMRON brand to measure the systolic blood pressure in 2 measurements with an interval of 2 min.

		Measurement 1 ± TD	Measurement 2 ± TD	*p* *
**Women with Hypertension diagnosis (*n* = 1106)**				
15–17 (*n* = 12)	Systolic blood pressure (mmHg)	113.42 ± 9.76	111.25 ± 8.69	0.031
18–29 (*n* = 126)	Systolic blood pressure (mmHg)	113.00 ± 13.05	110.66 ± 13.07	<0.001
30–39 (*n* = 194)	Systolic blood pressure (mmHg)	117.20 ± 16.73	114.67 ± 16.28	<0.001
40–49 (*n* = 261)	Systolic blood pressure (mmHg)	127.98 ± 21.65	126.00 ± 22.40	<0.001
50–59 (*n* = 343)	Systolic blood pressure (mmHg)	132.25 ± 21.66	129.85 ± 21.47	<0.001
60–69 (*n* = 428)	Systolic blood pressure (mmHg)	138.13 ± 23.01	135.24 ± 22.63	<0.001
70–79 (*n* = 287)	Systolic blood pressure (mmHg)	147.44 ± 26.00	144.64 ± 25.60	<0.001
80–89 (*n* = 151)	Systolic blood pressure (mmHg)	152.54 ± 25.10	150.00 ± 25.05	<0.001
>90 (*n* = 18)	Systolic blood pressure (mmHg)	147.06 ± 31.90	144.22 ± 32.67	0.055
**Women without Hypertension diagnosis (*n* = 17,414)**				
15–17 (*n* = 989)	Systolic blood pressure (mmHg)	108.23 ± 10.72	105.77 ± 10.34	<0.001
18–29 (*n* = 5451)	Systolic blood pressure (mmHg)	108.81 ± 10.44	106.90 ± 10.15	<0.001
30–39 (*n* = 4903)	Systolic blood pressure (mmHg)	111.30 ± 11.54	109.29 ± 11.25	<0.001
40–49 (*n* = 2555)	Systolic blood pressure (mmHg)	115.90 ± 14.19	113.96 ± 14.03	<0.001
50–59 (*n* = 1586)	Systolic blood pressure (mmHg)	120.36 ± 16.11	117.83 ± 16.10	<0.001
60–69 (*n* = 1053)	Systolic blood pressure (mmHg)	126.95 ± 18.53	124.34 ± 18.35	<.001
70–79 (*n* = 598)	Systolic blood pressure (mmHg)	130.87 ± 20.02	127.88 ± 19.80	<0.001
80–89 (*n* = 238)	Systolic blood pressure (mmHg)	136.67 ± 23.28	134.00 ± 23.39	<0.001
>90 (*n* = 41)	Systolic blood pressure (mmHg)	132.27 ± 26.33	129.88 ± 27.04	0.009

mmHg: millimeters of mercury; TD: Typical Deviation; * *p* values were calculated through paired samples test.

**Table 6:** Relative and absolute reliability indices for women with and without a diagnosis of HT.

Age Intervals (years)	ICC (95% CI)	SEM (mmHg)	%SEM	SRD (mmHg)	%SRD
**Women with Hypertension diagnosis (*n* = 1820)**					
15–17 (*n* = 12)	0.92 (0.77–0.98)	2.54	2.26	7.05	6.27
18–29 (*n* = 126)	0.92 (0.89–0.94)	3.65	3.26	10.11	9.04
30–39 (*n* = 194)	0.94 (0.93–0.96)	3.91	3.37	10.83	9.34
40–49 (*n* = 261)	0.96 (0.95–0.97)	4.41	3.47	12.21	9.62
50–59 (*n* = 343)	0.95 (0.94–0.96)	4.82	3.68	13.37	10.20
60–69 (*n* = 428)	0.94 (0.93–0.95)	5.4	3.95	14.96	10.95
70–79 (*n* = 287)	0.96 (0.95–0.97)	5.22	3.58	14.48	9.92
80–89 (*n* = 151)	0.96 (0.95–0.97)	4.69	3.10	13	8.6
<90 (*n* = 18)	0.98 (0.95–0.99)	4.45	3.06	12.34	8.47
**Women without Hypertension diagnosis (*n* = 17,414)**					
15–17 (*n* = 989)	0.87 (0.86–0.89)	3.75	3.51	10.4	9.72
18–29 (*n* = 5451)	0.88 (0.88–0.89)	3.54	3.73	9.8	9.09
30–39 (*n* = 4903)	0.89 (0.89–0.90)	3.74	3.4	10.38	9.41
40–49 (*n* = 2555)	0.92 (0.91–0.93)	3.99	3.47	11.06	9.62
50–59 (*n* = 1586)	0.93 (0.92–0.94)	4.29	3.6	11.9	9.99
60–69 (*n* = 1053)	0.94 (0.93–0.95)	4.48	3.57	12.42	9.88
70–79 (*n* = 598)	0.94 (0.93–0.95)	4.92	3.8	13.63	10.35
80–89 (*n* = 238)	0.96 (0.95–0.97)	4.73	3.49	13.1	9.68
<90 (*n* = 41)	0.97 (0.95–0.99)	4.22	3.22	11.7	8.92

ICC: intraclass correlation coefficient; CI: confidence interval; SEM: standard error of measurement; SRD: smallest real difference; mmHg: millimeters of mercury.

**Table 7:** MmHg in the digital monitor of the OMRON brand to measure the diastolic blood pressure in 2 measurements with an interval of 2 min.

		Measurement 1 ± TD	Measurement 2 ± TD	*p* *
All (*n* = 33,522)	Diastolic blood pressure (mmHg)	71.97 ± 10.07	71.21 ± 10.05	<0.001

mmHg: millimeters of mercury; TD: Typical Deviation; * *p* values were calculated through paired samples test.

**Table 8:** Diastolic blood pressure test–retest reliability of two measurements taken with a 2-min interval (*n* = 33,522).

ICC (95% CI)	SEM (mmHg)	%SEM	SRD (mmHg)	%SRD
0.94 (0.94–0.94)	2.53	3.53	7	9.78

ICC: intraclass correlation coefficient; CI: confidence interval; SEM: standard error of measurement; SRD: smallest real difference; mmHg: millimeters of mercury.

**Table 9:** MmHg in the digital monitor of the OMRON brand to measure the diastolic blood pressure in 2 measurements with an interval of 2 min.

		Measurement 1 ± TD	Measurement 2 ± TD	*p* *
**Men with Hypertension diagnosis (*n* = 1106)**				
15–17 (*n* = 6)	Diastolic blood pressure (mmHg)	68.83 ± 5.98	65.83 ± 4.87	0.068
18–29 (*n* = 48)	Diastolic blood pressure (mmHg)	74.92 ± 12.87	74.42 ± 11.40	0.371
30–39 (*n* = 107)	Diastolic blood pressure (mmHg)	81.09 ± 10.86	79.88 ± 10.37	0.002
40–49 (*n* = 181)	Diastolic blood pressure (mmHg)	82.03 ± 12.99	81.07 ± 12.62	<0.001
50–59 (*n* = 199)	Diastolic blood pressure (mmHg)	83.24 ± 12.68	82.31 ± 12.47	<0.001
60–69 (*n* = 220)	Diastolic blood pressure (mmHg)	79.45 ± 11.83	78.46 ± 11.22	<0.001
70–79 (*n* = 220)	Diastolic blood pressure (mmHg)	76.93 ± 12.50	76.51 ± 12.39	0.100
80–89 (*n* = 114)	Diastolic blood pressure (mmHg)	73.23 ± 12.88	72.92 ± 12.38	0.380
>90 (*n* = 11)	Diastolic blood pressure (mmHg)	80.55 ± 9.97	78.00 ± 9.85	0.034
**Men without Hypertension diagnosis (*n* = 13,133)**				
15–17 (*n* = 876)	Diastolic blood pressure (mmHg)	67.93 ± 8.00	66.42 ± 8.13	<0.001
18–29 (*n* = 3210)	Diastolic blood pressure (mmHg)	72.04 ± 9.23	70.82 ± 9.18	<0.001
30–39 (*n* = 3535)	Diastolic blood pressure (mmHg)	74.91 ± 9.10	74.20 ± 9.19	<0.001
40–49 (*n* = 2208)	Diastolic blood pressure (mmHg)	76.80 ± 10.03	76.33 ± 9.96	<0.001
50–59 (*n* = 1479)	Diastolic blood pressure (mmHg)	76.57 ± 9.80	75.96 ± 9.82	<0.001
60–69 (*n* = 1012)	Diastolic blood pressure (mmHg)	75.56 ± 10.34	74.87 ± 10.24	<0.001
70–79 (*n* = 541)	Diastolic blood pressure (mmHg)	73.28 ± 10.68	72.70 ± 10.82	<0.001
80–89 (*n* = 240)	Diastolic blood pressure (mmHg)	69.55 ± 10.68	68.78 ± 11.00	0.001
>90 (*n* = 32)	Diastolic blood pressure (mmHg)	68.28 ± 10.54	67.50 ± 11.11	0.212

mmHg: millimeters of mercury; TD: Typical Deviation; * *p* values were calculated through paired samples test.

**Table 10:** Relative and absolute reliability indices for men with and without a diagnosis of HT.

Age Intervals (years)	ICC (95% CI)	SEM (mmHg)	%SEM	SRD (mmHg)	%SRD
**Men with Hypertension diagnosis (*n* = 1106)**					
15–17 (*n* = 6)	0.73 (0.02–0,95)	2.24	4.22	7.88	11.7
18–29 (*n* = 48)	0.95 (0.91–0.97)	2.69	3.6	7.45	9.97
30–39 (*n* = 107)	0.93 (0.90–0.95)	2.85	3.5	7.9	9.81
40–49 (*n* = 181)	0.96 (0.95–0.97)	2.53	3.10	7.01	8.6
50–59 (*n* = 199)	0.96 (0.94–0.97)	2.61	3.15	7.23	8.73
60–69 (*n* = 220)	0.95 (0.93–0.96)	2.63	3.33	7.29	9.23
70–79 (*n* = 220)	0.95 (0.94–0.96)	2.64	3.44	7.32	9.54
80–89 (*n* = 114)	0.96 (0.94–0.97)	2.62	3.58	7.26	9.93
<90 (*n* = 11)	0.91 (0.72–0.98)	2.92	3.69	8.10	10.22
**Men without Hypertension diagnosis (*n* = 13,133)**					
15–17 (*n* = 876)	0.87 (0.85–0.88)	2.92	4.35	8.09	12.05
18–29 (*n* = 3210)	0.91 (0.91–0.92)	2.7	3.78	7.48	10.49
30–39 (*n* = 3535)	0.93 (0.92–0.93)	2.47	3.31	6.85	9.18
40–49 (*n* = 2208)	0.94 (0.94–0.95)	2.43	3.17	6.73	8.79
50–59 (*n* = 1479)	0.94 (0.93–0.95)	2.38	3.13	6.61	8.66
60–69 (*n* = 1012)	0.95 (0.94–0.95)	2.35	3.12	6.50	8.65
70–79 (*n* = 541)	0.95 (0.94–0.95)	2.5	3.42	6.92	9.49
80–89 (*n* = 240)	0.95 (0.93–0.96)	2.47	3.57	6.85	9.9
<90 (*n* = 32)	0.95 (0.90–0.97)	2.44	3.6	6.78	9.98

ICC: intraclass correlation coefficient; CI: confidence interval; SEM: standard error of measurement; SRD: smallest real difference; mmHg: millimeters of mercury.

**Table 11:** MmHg in the digital monitor of the OMRON brand to measure the diastolic blood pressure in 2 measurements with an interval of 2 min.

		Measurement 1 ± TD	Measurement 2 ± TD	*p* *
**Women with Hypertension diagnosis (*n* = 1106)**				
15–17 (*n* = 12)	Diastolic blood pressure (mmHg)	71.67 ± 6.72	72.75 ± 7.46	0.218
18–29 (*n* = 126)	Diastolic blood pressure (mmHg)	71.32 ± 9.47	70.21 ± 9.46	<0.001
30–39 (*n* = 194)	Diastolic blood pressure (mmHg)	74.48 ± 11.24	73.69 ± 11.62	0.001
40–49 (*n* = 261)	Diastolic blood pressure (mmHg)	79.64 ± 13.16	79.24 ± 12.90	0.073
50–59 (*n* = 343)	Diastolic blood pressure (mmHg)	77.06 ± 10.76	76.23 ± 10.52	<0.001
60–69 (*n* = 428)	Diastolic blood pressure (mmHg)	74.54 ± 11.03	73.79 ± 10.98	<0.001
70–79 (*n* = 287)	Diastolic blood pressure (mmHg)	73.26 ± 12.00	72.33 ± 12.34	<0.001
80–89 (*n* = 151)	Diastolic blood pressure (mmHg)	71.09 ± 10.96	70.15 ± 10.73	0.004
>90 (*n* = 18)	Diastolic blood pressure (mmHg)	69.33 ± 11.79	68.56 ± 11.37	0.353
**Women without Hypertension diagnosis (*n* = 17,414)**				
15–17 (*n* = 989)	Diastolic blood pressure (mmHg)	66.49 ± 8.49	65.24 ± 8.53	<0.001
18–29 (*n* = 5451)	Diastolic blood pressure (mmHg)	67.60 ± 8.36	66.83 ± 8.23	<0.001
30–39 (*n* = 4903)	Diastolic blood pressure (mmHg)	70.08 ± 8.95	69.48 ± 8.88	<0.001
40–49 (*n* = 2555)	Diastolic blood pressure (mmHg)	71.88 ± 9.41	71.37 ± 9.41	<0.001
50–59 (*n* = 1586)	Diastolic blood pressure (mmHg)	71.93 ± 9.59	71.22 ± 9.45	<0.001
60–69 (*n* = 1053)	Diastolic blood pressure (mmHg)	71.32 ± 9.36	70.65 ± 9.35	<0.001
70–79 (*n* = 598)	Diastolic blood pressure (mmHg)	69.82 ± 10.29	68.90 ± 10.22	<0.001
80–89 (*n* = 238)	Diastolic blood pressure (mmHg)	68.41 ± 10.59	67.67 ± 10.46	0.001
>90 (*n* = 41)	Diastolic blood pressure (mmHg)	68.02 ± 10.23	67.56 ± 10.85	0.318

mmHg: millimeters of mercury; TD: Typical Deviation; * *p* values were calculated through paired samples test.

**Table 12:** Relative and absolute reliability indices for women with and without a diagnosis of HT.

Age Intervals (years)	ICC (95% CI)	SEM (mmHg)	%SEM	SRD (mmHg)	%SRD
**Women with Hypertension diagnosis (*n* = 1820)**					
15–17 (*n* = 12)	0.91 (0.74–0.97)	2.09	2.9	5.8	8.03
18–29 (*n* = 126)	0.93 (0.90–0.95)	2.47	3.49	6.84	9.67
30–39 (*n* = 194)	0.96 (0.94–0.97)	2.37	3.2	6.57	8.87
40–49 (*n* = 261)	0.96 (0.95–0.97)	2.57	3.24	7.13	8.98
50–59 (*n* = 343)	0.95 (0.94–0.96)	2.4	3.13	6.66	8.69
60–69 (*n* = 428)	0.95 (0.94–0.96)	2.53	3.42	7.02	9.47
70–79 (*n* = 287)	0.96 (0.94–0.96)	2.55	3.51	7.08	9.72
80–89 (*n* = 151)	0.93 (0.90–0.95)	2.87	4.06	7.96	11.27
<90 (*n* = 18)	0.96 (0.89–0.98)	2.43	3.52	6.73	9.77
**Women without Hypertension diagnosis (*n* = 17,414)**					
15–17 (*n* = 989)	0.89 (0.88–0.91)	2.76	4.19	7.65	11.61
18–29 (*n* = 5451)	0.90 (0.90–0.91)	2.56	3.81	7.09	10.55
30–39 (*n* = 4903)	0.92 (0.92–0.93)	2.46	3.52	6.81	9.76
40–49 (*n* = 2555)	0.93 (0.93–0.94)	2.4	3.35	6.65	9.29
50–59 (*n* = 1586)	0.94 (0.93–0.94)	2.39	3.34	6.63	9.26
60–69 (*n* = 1053)	0.93 (0.92–0.94)	2.48	3.49	6.86	9.67
70–79 (*n* = 598)	0.94 (0.93–0.95)	2.47	3.56	6.85	9.87
80–89 (*n* = 238)	0.95 (0.93–0.96)	2.38	3.49	6.59	9.68
<90 (*n* = 41)	0.96 (0.93–0.98)	2.08	3.07	5.77	8.51

## Data Availability

The datasets used during the current study are available from the corresponding author on reasonable request.
